# Association of interferon regulatory factor 4 gene polymorphisms rs12203592 and rs872071 with skin cancer and haematological malignancies susceptibility: a meta-analysis of 19 case–control studies

**DOI:** 10.1186/1471-2407-14-410

**Published:** 2014-06-06

**Authors:** Songtao Wang, Qing Yan, Pin Chen, Peng Zhao, Aihua Gu

**Affiliations:** 1 Department of Neurosurgery, The First Affiliated Hospital, Nanjing Medical University, Nanjing, China; 2 State Key Laboratory of Reproductive Medicine, Institute of Toxicology, Nanjing Medical University, Nanjing, China; 3 Key Laboratory of Modern Toxicology of Ministry of Education, School of Public Health, Nanjing Medical University, Nanjing, China

**Keywords:** Meta-analysis, IRF4, Interferon regulatory factor 4, Polymorphisms, rs12203592, rs872071, Cancer risk

## Abstract

**Background:**

Research has indicated that the rs12203592 and rs872071 interferon regulatory factor 4 (IRF4) gene polymorphisms correlate with the risk of cancer, especially skin cancer and haematological malignancies, but the results remain controversial. To understand better the effects of these two polymorphisms on skin cancer and haematological malignancies susceptibility, a cumulative meta-analysis was performed.

**Methods:**

We conducted a search using the PubMed and Web of Science databases for relevant case-control studies published before April 2014. Summary odds ratios (ORs) and corresponding 95% confidence intervals (CIs) were estimated using fixed- or random-effects models where appropriate. Heterogeneity test, publication bias test, and sensitivity analysis were also performed.

**Results:**

In total, 11 articles comprised of 19 case–control studies were identified; five focused on the rs12203592 polymorphism with 7,992 cases and 8,849 controls, and six were on the rs872071 polymorphism with 3108 cases and 8300 controls. As for rs12203592, a significant correlation with overall skin cancer and haematological malignancies risk was found with the homozygote comparison model (OR = 1.566, 95% CI 1.087-2.256) and recessive model (OR = 1.526, 95% CI 1.107-2.104). For rs872071, a significantly elevated haematological malignancies risk was observed in all genetic models (homozygote comparison: OR = 1.805, 95% CI 1.402-2.323; heterozygote comparison: OR = 1.427, 95% CI 1.203-1.692; dominant: OR = 1.556, 95% CI 1.281-1.891; recessive: OR = 1.432, 95% CI 1.293-1.587; additive: OR = 1.349, 95% CI 1.201-1.515). Similarly, increased skin cancer and haematological malignancies risk was also identified after stratification of the SNP data by cancer type, ethnicity and source of controls for both polymorphisms.

**Conclusions:**

Our meta-analysis indicated that the rs12203592 and rs872071 IRF4 gene polymorphisms are associated with individual susceptibility to skin cancer and haematological malignancies. Moreover, the effect of the rs12203592 polymorphism on skin cancer risk was particularly prominent among Caucasians. Further functional research should be performed to validate the association.

## Background

Cancer is a multifactorial disease resulting from complex interactions between environmental and genetic factors. Cancer is one of the leading causes of death worldwide [1,2]. Skin cancer is the most common carcinoma, affecting millions worldwide [3]. The two major groups of skin cancer are non-melanoma and melanoma. The most common type of non-melanoma skin cancer is basal cell carcinoma (BCC) followed by squamous cell carcinoma (SCC). Melanoma is a malignant tumour of melanocytes. Melanoma can occur in any part of the body and is thought to be the most fatal form of skin cancer [4-7].

Haematological malignancies, including leukaemia, lymphoma and plasma cell dyscrasia, are a group of disorders that affect blood, bone marrow, lymph nodes and spleen. These malignancies make up approximately 9.5% of all new cancer diagnoses in the United States [8,9]. Haematological malignancies, such as chronic lymphocytic leukaemia (CLL), multiple myeloma(MM), Hodgkin lymphoma (HL) and non-Hodgkin lymphoma (NHL), are derived from lymphocyte cells [10,11]. Among these neoplasms, CLL is the most common form of lymphoid malignancy in Western countries [12]; malignant lymphoma, generally divided into HL and NHL, is the most common haematological malignancy in the world. MM is a malignancy of plasma cells with a complex aetiology [13,14]. However, the exact mechanism of carcinogenesis remains unclear. In recent years, evidence has revealed that genetic variation can modulate several important biological processes, thereby altering cancer susceptibility.

Interferon regulatory factors (IRF) are a family of transcription factors characterised by a DNA-binding domain containing a five-tryptophan residue repeat [15,16]. IRFs are widely expressed and regulate not only the cellular response to interferons but also cell growth, susceptibility to transformation by oncogenes, induction of apoptosis, and the development of the T-cell immune response [17]. The IRF family contains at least ten proteins that modulate the expression of interferon-inducible genes, considered to play an important role in the immune response and tumorigenesis [15,18,19]. As a member of the IRF family of transcription factors, IRF4 (also known as multiple myeloma 1 (MUM1) and lymphocyte-specific interferon regulatory factor (LSIRF)) is expressed in most cell types of the immune system [20,21]. Recently, IRF4 was reported to be essential to the development and function of T helper (Th) cells, regulatory T (Treg) cells, B cells and dendritic cells [22,23]. Research has demonstrated that IRF4 plays a pivotal role in the development and progression of cancer, particularly in skin cancer and haematopoietic malignancies [22,24].

Genome-wide association studies (GWAS) are the ideal strategy to select common, low-penetrance susceptibility loci without prior hypotheses about the role of the genes in disease development. Recent GWAS have reported that variants at several sites within the IRF4 gene may be implicated in the risk of cancer. Most importantly, the intron 4 SNP rs12203592 and 3′ UTR SNP rs872071 are associated with an increased risk for melanoma, BCC [25,26], CLL and MM [27,28]. Given that the IRF4 gene has been recognised as one of the most common tumour markers, numerous studies have assessed the possible association between the IRF4 polymorphisms and cancer risk. However, the results are inconclusive. To derive a more precise estimation of the relationship between the rs12203592 and rs872071 IRF4 polymorphisms and cancer risk, we performed a meta-analysis of all available case-control studies.

## Methods

### Literature search strategy

We searched the PubMed and Web of Science databases for all relevant articles regarding IRF4 SNPs associated with cancer risk (search last updated on April 10, 2014). The following keywords were used: “IRF4” or “interferon regulatory factor 4”, “polymorphisms or variant or SNP or mutation” and “cancer or tumor or neoplasm or carcinoma”. The search was conducted exclusively on human subjects. The reference lists of reviews and retrieved articles were simultaneously hand-searched. We did not consider abstracts or unpublished reports.

### Inclusion criteria

All abstracts of citations and retrieved studies were reviewed. The following criteria were used to identify eligible published studies: (i) the study evaluated the association between the IRF4 polymorphisms (rs12203592 and rs872071) and cancer risk; (ii) the publication was a case–control or case-cohort study; (iii) the paper provided sample size, distribution of alleles, genotypes or other information that can help us estimate an OR with 95% confidence interval (95% CI); (iv) the genotype distribution of the control population is consistent with Hardy-Weinberg Equilibrium (HWE). Accordingly, publications were excluded using the following criteria: (i) articles that were not about cancer research; (ii) the publication contained duplicated previous research; (iii) the study did not include usable genotype data were excluded.

### Data extraction

Two investigators independently extracted information from all eligible publications according to the inclusion criteria listed above. The results were compared, and disagreements were resolved by discussion until a consensus was reached. Data extracted from each study included the following characteristics: the first author’s name, the year of publication, the country of participants, ethnicity, cancer type, source of control group (population- or hospital-based controls), and the genotype frequency of the rs12203592 and rs872071 polymorphism in the cases and controls.

### Statistical analysis

All statistical analysis was performed using STATA software (version 11.0; STATA Corporation, College Station, TX). Two sided P-values < 0.05 were considered statistically significant. Our meta-analysis recalculated HWE in the controls for each study. The goodness of fit test (chi-square or Fisher’s exact test) was used to assess deviation from HWE (significant at the 0.05 level). Studies that deviated from HWE were removed.

ORs with 95% CIs were used to estimate the strength of the association between the IRF4 rs12203592 and rs872071 polymorphisms and skin cancer and haematopoietic malignancies risk. In addition, the Z-test was also used, and a P value < 0.05 indicated statistical significance for the association. We examined the association between the rs12203592 IRF4 polymorphism and skin cancer and haematopoietic malignancies using the homozygote comparison (TT versus CC), heterozygote comparison (CT versus CC), dominant genetic model (TT + CT versus CC), recessive genetic model (TT versus CT + CC) and additive genetic model (T versus C). The same methods were applied to the analysis of the rs872071 IRF4 polymorphism. In addition, a stratified analysis was also performed based on cancer type, ethnicity and the source of controls.

The assumption of heterogeneity was ascertained using a chi-based Q-test. A P-value less than 0.05 for the Q test indicated significant heterogeneity among the studies. The pooled OR was estimated using a fixed- or random-effects model, where appropriate. If the P value was less than 0.05 indicative of heterogeneity across studies, a random-effects model (DerSimonian and Laird) was utilised for the meta-analysis [29]. Otherwise, a fixed-effect model (Mantel-Haenszel) was used [30]. We also quantified the effect of heterogeneity using the *I*^2^ test. *I*^2^ values of 25, 50, and 75% were indicative of low, moderate, and high heterogeneity, respectively.

The sensitivity analysis was conducted by removing one study at a time to evaluate the quality and consistency of the meta-analysis results. Publication bias was qualitatively and quantitatively assessed using the Begg’s funnel plots and Egger’s test, respectively [31]. To ensure the reliability and the accuracy of the results, two reviewers independently assessed the data using the statistical software programmes and obtained the same results.

## Results

### Literature search and characteristics

**Figure 1:**
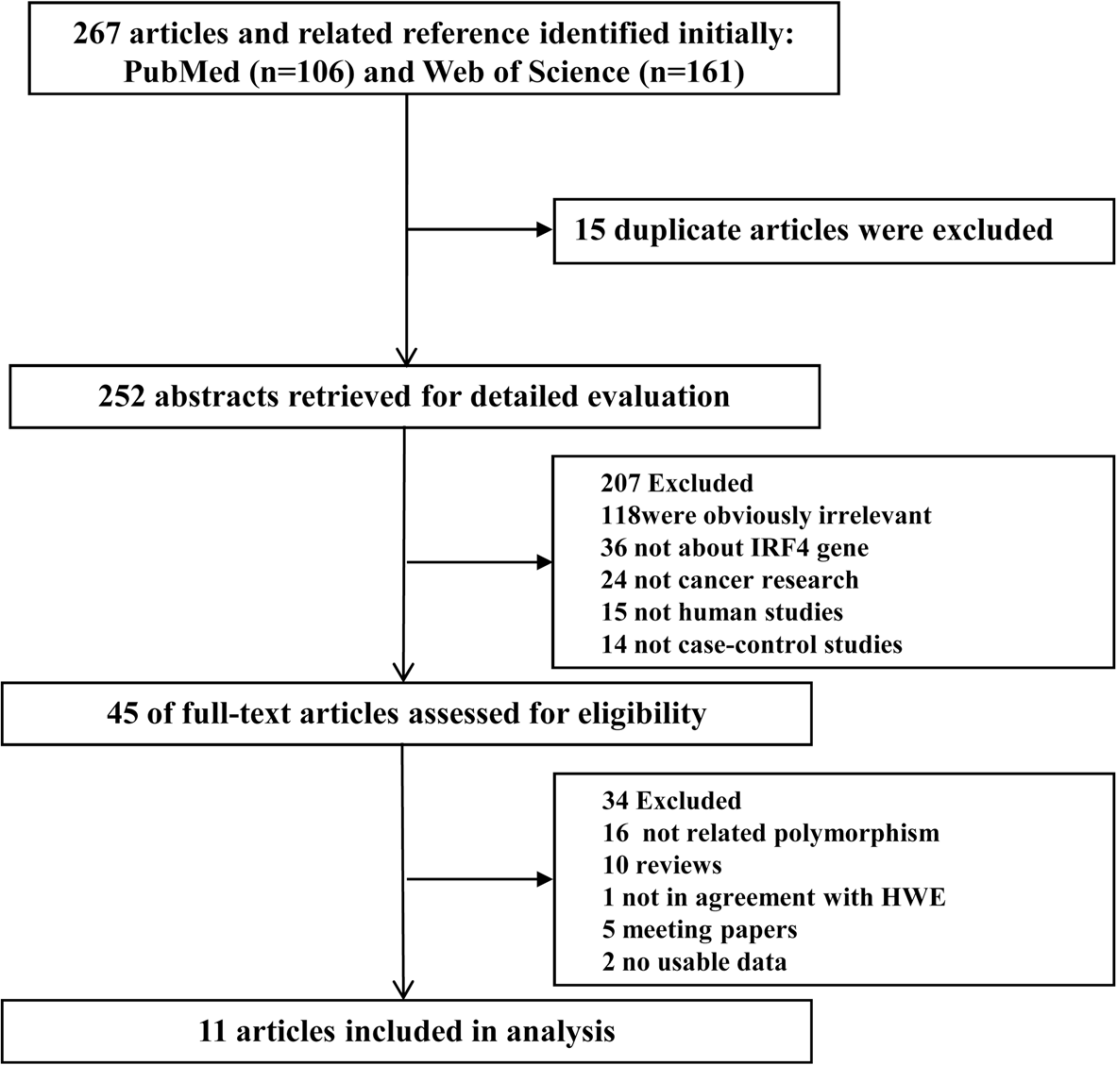
Flow chart of literature search and study selection.

A total of 179 potential individual publications were initially identified after a systematic literature search of the PubMed, Embase and Web of Science databases. The titles, abstracts and full texts of the retrieved articles were reviewed based on the inclusion criteria shown in Figure 1. Finally, we identified 11 eligible articles comprised of 19 studies for the current meta-analysis [24,27,32-40]. In all the eligible articles, studies by Han et al., Wang et al., Broderick et al., Di Bernardo et al. include different sets of data, and each set of data was treated as a separate case–control study in this meta-analysis (Table 1). Our meta-analysis included 11 of the rs12203592 polymorphism with 7,992 cases and 8,849 controls and 8 studies of the rs872071 polymorphism with 3108 cases and 8300 controls. All studies were published in English. The control genotype distributions of all studies were in accordance with HWE. Detailed study characteristics included in the current meta-analysis are presented in Table 1.

**Table 1:** Main characteristics of all studies included in the meta-analysis

**rs12203592**						**Case**			**Control**			
**First author**	**Year**	**Country**	** Ethnicity**	**Cancer type**	**Source of controls**	**CC**	**TC**	**TT**	**CC**	**TC**	**TT**	**HWE**
Gathany [24]	2009	USA	Mixed	NHL	PB	730	239	19	605	202	18	0.814
Wang 1 [40]	2009	USA	Mixed	NHL	PB	299	123	13	357	143	15	0.882
Wang 2 [40]	2009	USA	Mixed	NHL	PB	332	157	29	283	160	19	0.541
Kvaskoff [34]	2011	France	Caucasians	Melanoma	HB	654	307	48	871	518	79	0.862
Han 1 [35]	2011	USA	Caucasians	Melanoma	HB	123	54	11	450	163	19	0.369
Han 2 [35]	2011	USA	Caucasians	Melanoma	HB	126	68	20	565	232	29	0.394
Pena-Chilet [32]	2013	Spain	Caucasians	Melanoma	PB	398	121	19	242	101	19	0.055
Han 3 [35]	2011	USA	Caucasians	SCC	HB	156	80	15	450	163	19	0.369
Han 4 [35]	2011	USA	Caucasians	SCC	HB	143	110	20	565	232	29	0.394
Han 5 [35]	2011	USA	Caucasians	BCC	HB	139	54	20	503	191	24	0.269
Han 6 [35]	2011	USA	Caucasians	BCC	HB	174	81	19	565	232	29	0.394
**rs872071**						**Case**			**Control**			
**First author**	**Year**	**Country**	** Ethnicity**	**Cancer type**	**Source of controls**	**AA**	**AG**	**GG**	**AA**	**AG**	**GG**	**HWE**
Di Bernardo 1 [27]	2008	UK	Caucasians	CLL	HB	63	248	190	305	720	411	0.754
Di Bernardo 2 [27]	2008	UK	Caucasians	CLL	PB	67	232	201	183	391	202	0.816
Lan [36]	2010	USA	Asians	CLL	HB	21	36	12	602	534	127	0.978
Crowther-Swanepoel [37]	2010	UK	Caucasians	CLL	PB	74	203	117	129	179	89	0.076
Broderick 1 [39]	2010	UK	Caucasians	HL	PB	16	55	36	228	519	278	0.629
Broderick 2 [39]	2010	UK	Caucasians	HL	PB	83	198	118	259	562	269	0.302
Pratt [38]	2010	UK	Caucasians	MM	PB	82	169	101	183	388	200	0.846
Qiao [33]	2013	China	Asians	NHL	HB	341	381	64	777	653	112	0.112

*BCC*: Basal cell carcinoma; *SCC*: Squamous cell carcinoma; *HL*: Hodgkin lymphoma; *NHL*: Non-Hodgkin lymphoma; *MM*: Multiple myeloma; *CLL*: Chronic lymphocytic leukaemia.

*PB*: population based; *HB*: hospital based.

*HWE*: Hardy-Weinberg equilibrium.

### Quantitative assessment of the included studies

**Figure 2:**
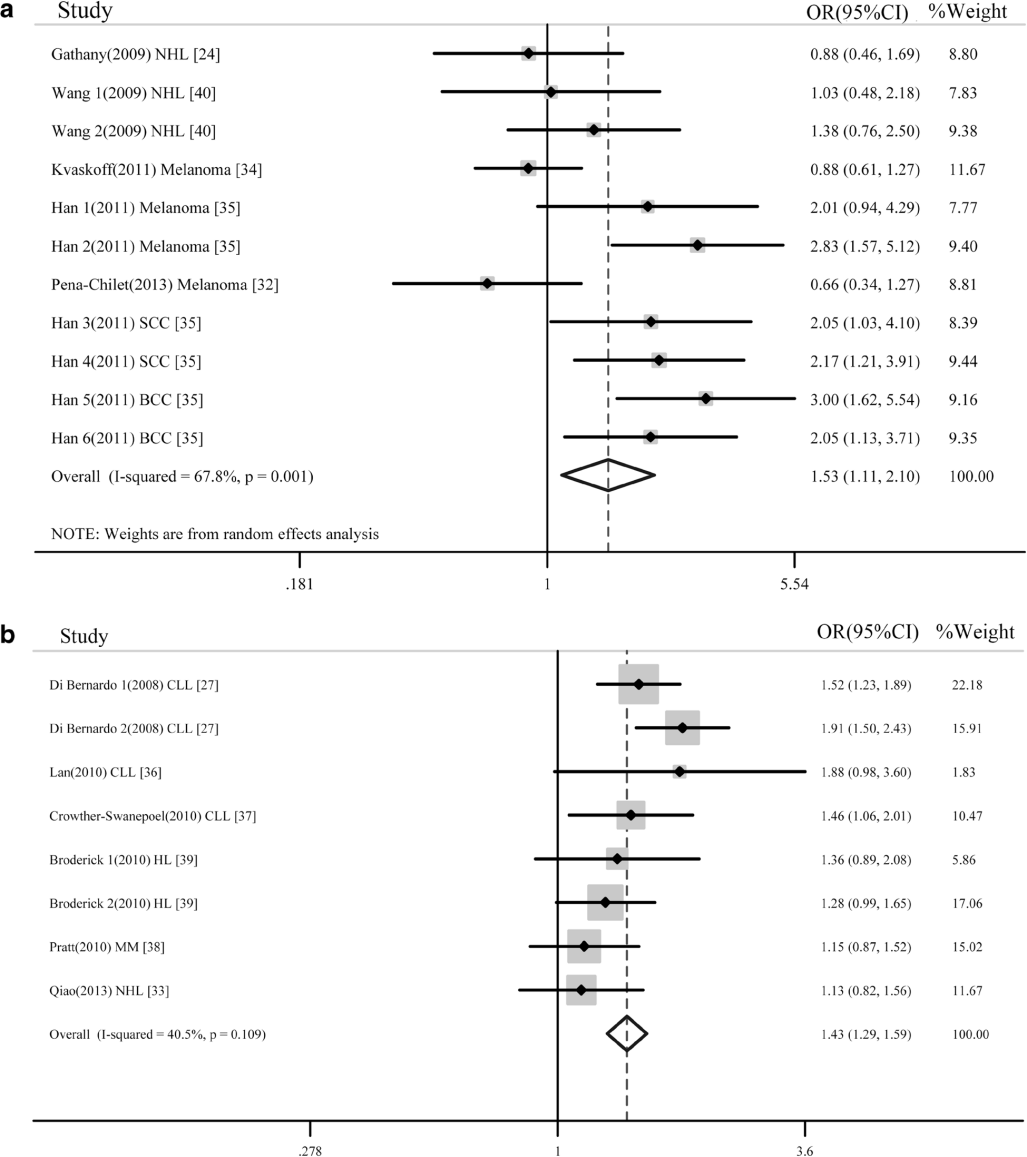
**Forest plots of ORs with 95% CIs for IRF4 polymorphisms and cancer susceptibility in the recessive model. (a)**. rs12203592, TT versus CT + CC. **(b)**. rs872071, GG versus GA+AA.

A summary of the meta-analysis findings on the association between the rs12203592 and rs872071 IRF4 polymorphisms and skin cancer and haematopoietic malignancies risk is presented in Table 2. With respect to the rs12203592 polymorphism, a total of 11 studies from 5 articles were included in this meta-analysis. Among the 11 studies, 8 focused on skin cancer and 3 on NHL. Eight studies used Caucasian populations, and 3 were from USA with mixed ethnicity. Four studies used population-based controls, and 7 used hospital-based controls. The overall analyses suggested a significant association between the rs12203592 polymorphism and skin cancer and haematopoietic malignancies susceptibility in the homozygote comparison model (OR = 1.566, 95% CI 1.087–2.256) and recessive model (OR = 1.526, 95% CI 1.107–2.104, Figure 2a) using the random-effects model. The results from the other genetic models were not significant. Specific data for the rs12203592 polymorphism were stratified by cancer type into the NHL subgroup or the skin cancer subgroup (melanoma, BBC and SCC). For skin cancer, a significantly increased risk was observed using the homozygote comparison model (OR = 1.728, 95% CI 1.145-2.608) and recessive model (OR = 1.808, 95% CI 1.127-2.900). However, no significant association was observed in the NHL subgroup. In the subgroup analysis stratified by ethnicity, a significantly elevated cancer risk was found among Caucasians with the homozygote comparison model (OR = 1.566, 95% CI 1.087–2.256) and recessive model (OR = 1.526, 95% CI 1.107–2.104) but not among Asian populations with all genetic models. When stratified by the source of controls, a significantly increased risk was observed in the hospital-based studies under four genetic models (homozygote comparison: OR = 2.094, 95% CI 1.314-3.336; dominant genetic model: OR = 1.314, 95% CI 1.002-1.723; recessive genetic model: OR = 1.959, 95% CI 1.310-2.931; additive model OR = 1.35, 95%CI 1.058 -1.725), whereas no significant association was observed in the population-based studies for all five genetic models.

**Table 2:** Main results of pooled ORs and stratification analysis of IRF4 polymorphisms on cancer risk in the meta-analysis

**rs12203592**	**TT vs CC**			**CT vs CC**			**Dominant model**			**Recessive model**			**Additive model**		
**C/T**	**OR**	**95% CI**	**P**^**a**^	**OR**	**95% CI**	**P**^**a**^	**OR**	**95% CI**	**P**^**a**^	**OR**	**95% CI**	**P**^**a**^	**OR**	**95% CI**	**P**^**a**^
**Overall**	**1.566**	(1.087,2.256)*	0.000	1.070	(0.904,1.266)*	0.000	1.135	(0.941,1.368)*	0.000	**1.526**	(1.107,2.104)*	0.001	1.168	(0.981,1.392)*	0.000
**Source of controls**															
PB	0.933	(0.672,1.295)	0.403	0.904	(0.793,1.031)	0.320	0.907	(0.799,1.029)	0.265	0.971	(0.701,1.345)	0.420	0.926	(0.830,1.033)	0.215
HB	**2.094**	(1.314,3.336)*	0.000	1.203	(0.936,1.547)*	0.000	**1.314**	(1.002,1.723)*	0.000	**1.959**	(1.310,2.931)*	0.003	**1.351**	(1.058,1.725)*	0.000
**Cancer type**															
Skin cancer	**1.808**	(1.127,2.900)*	0.000	1.130	(0.890,1.435)*	0.000	1.217	(0.935,1.584)*	0.000	**1.728**	(1.145,2.608)*	0.000	1.253	(0.983,1.596)*	0.000
Melanoma	1.315	(0.624,2.770)*	0.000	0.957	(0.724,1.265)*	0.009	1.011	(0.715,1.431)*	0.000	1.321	(0.684,2.552)*	0.001	1.057	(0.747,1.497)*	0.000
BCC	**2.511**	**(1.630,3.867)**	0.430	1.085	(0.861,1.368)	0.668	**1.245**	**(1.004,1.543)**	0.996	**2.451**	**(1.600,3.755)**	0.383	**1.352**	**(1.131,1.617)**	0.644
SCC	**2.520**	**(1.598,3.974)**	0.703	**1.651**	**(1.330,2.049)**	0.208	**1.743**	**(1.418,2.143)**	0.207	**2.120**	**(1.354,3.318)**	0.901	**1.643**	**(1.385,1.949)**	0.303
NHL	1.074	(0.734,1.571)	0.677	0.948	(0.820,1.096)	0.545	0.960	(0.834,1.104)	0.734	1.103	(0.756,1.608)	0.589	0.979	(0.867,1.106)	0.914
**Ethnicity**															
Caucasian	**1.808**	(1.127,2.900)*	0.000	1.130	(0.890,1.435)*	0.000	1.135	(0.941,1.368)*	0.000	**1.728**	(1.145,2.608)*	0.000	1.253	(0.983,1.596)*	0.000
Mixed	1.074	(0.734,1.571)	0.677	0.948	(0.820,1.096)	0.545	0.960	(0.834,1.104)	0.734	1.103	(0.756,1.608)	0.589	0.979	(0.867,1.106)	0.914
**rs872071**	**GG vs AA**			**GA vs AA**			**Dominant model**			**Recessive model**			**Additive model**		
**G/A**	**OR**	**95% CI**	**P**^**a**^	**OR**	**95% CI**	**P**^**a**^	**OR**	**95% CI**	**P**^**a**^	**OR**	**95% CI**	**P**^**a**^	**OR**	**95% CI**	**P**^**a**^
**Overall**	**1.805**	(1.402,2.323)*	0.002	**1.427**	(1.203,1.692)*	0.034	**1.556**	(1.281,1.891)*	0.003	**1.432**	**(1.293,1.587)**	0.109	**1.349**	(1.201,1.515)*	0.002
**Source of controls**															
PB	**1.767**	(1.244,2.511)*	0.003	**1.369**	(1.041,1.801)*	0.018	**1.478**	(1.278,1.708)*	0.003	**1.443**	**(1.270,1.639)**	0.070	**1.329**	(1.116,1.582)*	0.002
HB	**1.884**	(1.212,2.930)*	0.037	**1.446**	**(1.247,1.678)**	0.255	**1.637**	**(1.219,2.198)**	0.058	**1.414**	**(1.191,1.678)**	0.215	**1.333**	**(1.213,1.465)**	0.069
**Cancer type**															
CLL	**2.424**	**(1.995,2.945)**	0.841	**1.752**	**(1.466,2.093)**	0.825	**1.982**	**(1.674,2.346)**	0.968	**1.646**	**(1.431,1.892)**	0.445	**1.543**	**(1.407,1.692)**	0.647
Lymphoma	**1.396**	**(1.123,1.735)**	0.614	**1.279**	**(1.103,1.483)**	0.472	**1.308**	**(1.135,1.507)**	0.579	**1.245**	**(1.039,1.491)**	0.754	**1.206**	**(1.095,1.328)**	0.764
**Ethnicity**															
Caucasian	**1.844**	(1.377,2.470)*	0.003	**1.418**	(1.124,1.787)*	0.019	**1.567**	(1.214,2.023)*	0.003	**1.463**	**(1.311,1.633)**	0.115	**1.350**	(1.174,1.554)*	0.003
Asian	**1.451**	**(1.072,1.965)**	0.075	**1.380**	**(1.164,1.636)**	0.205	**1.390**	**(1.180,1.637)**	0.109	1.233	(0.924,1.646)	0.167	**1.256**	**(1.110,1.420)**	0.071

*PB*: population based; *HB*: hospital based; Bold data represent the positive results.

*Random-effects model was used when P value for heterogeneity test < 0.05; otherwise, fix-effects model was used.

^a^P value of Q-test for heterogeneity test; *BCC*: Basal cell carcinoma; *SCC*: Squamous cell carcinoma; *HL*: Hodgkin lymphoma; *NHL*: Non-Hodgkin lymphoma; *MM*: Multiple myeloma; *CLL*: Chronic lymphocytic leukaemia.

With respect to the rs872071 polymorphism, a total of 8 studies from 6 articles were included. Of the 8 eligible studies, 4 focused exclusively on CLL, 3 on lymphoma and 1 on MM. Caucasian populations were used in 6 studies, and Asians were assessed in 2. Five of the studies were population-based controls, and 3 used hospital-based controls. Overall, a significantly elevated haematological malignancies risk was associated with the rs872071 polymorphism in all genetic models (homozygote comparison: OR = 1.805, 95% CI 1.402-2.323; heterozygote comparison: OR = 1.427, 95% CI 1.203-1.692; dominant: OR = 1.556, 95% CI 1.281-1.89; recessive: OR = 1.432, 95% CI 1.293-1.587, Figure 2b; additive: OR = 1.349, 95% CI 1.201-1.515). After stratification by cancer type (HL and NHL were merged as lymphoma) and source of controls, a significant association was also observed with all of the genetic models. When we stratified the studies by ethnicity, a significant association was also observed in Caucasians under all genetic models. In Asians, the association was significant in all genetic models except for the recessive genetic model (OR = 1.233, 95% CI 0.924-1.646). The meta-analysis results for the subgroups are listed in Table 2.

### Test of heterogeneity

For the rs12203592 polymorphism, all genetic models showed significant heterogeneity. After subgroup analysis by cancer type, the heterogeneity was effectively removed in the NHL subgroup. In the analysis of ethnicity, the heterogeneity significantly disappeared in the mixed subgroup. When stratified based on the source of controls, the heterogeneity also disappeared in the population-based control subgroups. The heterogeneity values are presented in Table 2.

Significant heterogeneities were observed in the overall analysis of the association between the rs872071 polymorphism and haematological malignancies risk in four genetic models (homozygote comparison, heterozygote comparison, dominant genetic model and additive model). After subgroup analysis by cancer type, the heterogeneity effectively disappeared in the CLL subgroup and lymphoma subgroup. In the analysis of ethnicity, the heterogeneity was significantly removed in the Asian population but remained in the Caucasians. When stratified based on the source of controls, heterogeneity was not observed in the hospital-based control subgroups. The heterogeneity values are presented in Table 2.

### Publication bias and sensitivity analysis

**Figure 3:**
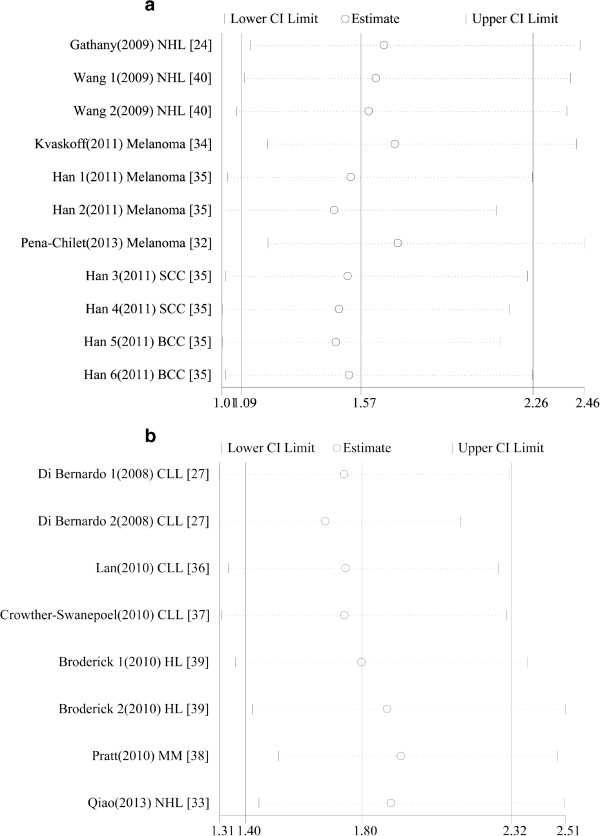
**Results of the sensitivity analysis examining the association between the IRF4 and cancer risk polymorphisms and cancer risk in homozygote comparison model. (a)**. rs12203592, TT versus CC. **(b)**. rs872071, GG versus AA.

Publication biases of the literature were investigated using the Begg’s funnel plot and Egger’s test. With respect to the rs12203592 polymorphism, the results of the Begg’s funnel plot suggested no publication bias. The Egger’s test did not show statistical evidence for publication bias (homozygote comparison model: t = 1.38, P = 0.201). The shapes of the funnel plots did not reveal any evidence of obvious asymmetry for the rs872071 polymorphism with all genetic models (Figure not shown). Similarly, the results of the Egger’s test indicated a lack of publication bias (homozygote comparison model: t =0.61, P = 0.556).Sensitivity analyses were also performed in the current meta-analysis to assess the influence of each individual study on the pooled ORs by sequential removal of individual studies. For both the rs12203592 and rs872071 polymorphisms in the IRF4 gene, the results suggested that no individual study significantly altered the pooled results, thereby suggesting that the results of this meta-analysis are robust and reliable (Figure 3a and 3b).

## Discussion

IRF4, a member of the IRF family of transcription factors, is expressed in cells of the immune system and transduces signals from various receptors to activate or suppress gene expression [15,41]. The product of the IRF4 gene is confined to cells of the immune system and melanocytic lineages [42]. It is considered a key regulator of several steps in lymphoid-, myeloid-, and dendritic-cell differentiation. It promotes the differentiation of mature B cells into antibody-secreting plasma cells [43,44]. Additionally, research has revealed that the IRF4 protein is expressed in a wide spectrum of haematological malignancies and skin cancers [42,45]. IRF4 plays an important role in cancer pathogenesis and acts as a potential marker for haematological neoplasms and malignant melanoma [19,45]. Recent GWAS findings have indicated that variants of the IRF4 gene were associated with the susceptibility to some cancer types, including CLL, HL, NHL, MM and skin cancer [25-27,35]. Given the possible of the IRF4 gene product in the immune response and carcinogenesis, numerous investigators have studied the possible association between the IRF4 polymorphisms and cancer risk, but the results are somewhat inconclusive. Meta-analysis is a powerful statistical method to combine comparable studies to increase the sample size and statistical power, thereby allowing a more compelling result to be drawn [46]. These advantages encouraged us to conduct this meta-analysis of all published articles investigating the association between IRF4 gene polymorphisms and cancer risk. To our knowledge, this is the first comprehensive meta-analysis examining the association of two common 6p25 variations (rs12203592 and rs872071) and skin cancer and haematological malignancies susceptibility.

In this meta-analysis, we included 11 eligible articles comprised of 19 case–control or cohort studies to explore the association between the rs12203592 and rs872071 IRF4 polymorphisms and skin cancer and haematological malignancies risk. We also performed subgroup analyses stratified by cancer type, ethnicity and source of controls. With respect to the rs12203592 polymorphism, 4901 cases and 5808 controls were included in the current meta-analysis. The pooled analyses suggested a significant association between the rs12203592 polymorphism and cancer susceptibility. In the subgroup analysis based on cancer type, a significant association was observed between this polymorphism and cancer risk exclusively in the skin cancer subgroup. However, no significant association between the rs12203592 polymorphism and NHL was found, indicating that the polymorphism may not be an independent risk factor for the development of NHL. When stratifying for ethnicity, a significant association was observed in Caucasian populations but not in other populations, suggesting genetic diversity among different ethnicities. Additionally, after stratification based on the source of controls, significantly increased risks were found in the hospital-based studies but not in the population-based studies. For the rs872071 polymorphism, 3108 cases and 8300 controls were included. Overall, there was evidence of an association between an increased risk of haematological malignancies and the rs872071 polymorphism in all genetic models when all of the eligible studies were pooled into the meta-analysis. In the subgroup analyses by cancer type and source of controls, an increased haematological malignancies risk was also observed in all genetic models. When stratified by ethnicity, a significantly increased risk was also observed for Asian populations in all of the genetic models except for the recessive model.

Heterogeneity is a potential problem when explaining the results of all meta-analyses. In the current study, the Q-test and *I*^2^ statistic were performed to test the significance of heterogeneity. For the rs12203592 polymorphism, significant heterogeneity was found in all comparison models. When stratified according to cancer type, ethnicity and source of controls, significant heterogeneity reduced or disappeared. For the rs872071 polymorphism, significant heterogeneity was detected in the overall comparisons. After subgroup analysis by cancer type, the heterogeneity was effectively decreased or removed. However, significant heterogeneity still existed Caucasian populations in certain genetic models when stratified according to ethnicity. This finding could be attributed to the fact that different genetic backgrounds and environments exist among different ethnicities and individuals. When stratified for the source of controls, significant heterogeneity still existed in some genetic models for the population-based studies. In this meta-analysis, the Begg’s funnel plot and Egger’s test were calculated to evaluate publication bias. Both the shape of the funnel plots and statistical results did not suggest publication bias. We also performed sensitivity analysis that indicated the results were reliable.

Several limitations in our meta-analysis should be acknowledged. First, the meta-analysis was based on the aggregation of published studies; unpublished data, ongoing studies and published articles were excluded. Studies with negative findings may have biased our results. Second, the number of cases and controls and small sample sizes, which could potentially influence the overall outcome, limited this meta-analysis. Third, most of the enrolled subjects were Caucasians and Asians; the populations of other races were under-represented. Fourth, due to the deficient adjusted data, we computed raw relative risks (RRs) from frequency distributions reported in the original publications, so our analyses are not adjusted for the main risk factors of both skin cancer and haematological malignancies. In addition, cancer is a complex disease with a multifactorial aetiology. Lack of original data for gene-gene and gene-environment interactions limited our further evaluation. Despite these limitations, the advantages of our meta-analysis should also be noted. First, studies that satisfactorily met our selection criteria were included in the present meta-analysis. The substantial number of cases and controls pooled from the different studies significantly increased the statistical power of the analysis. Second, the distribution of genotypes in the controls was in agreement with Hardy-Weinberg equilibrium (P > 0.05) for all studies. Third, the results of the Funnel plot and Egger’s test detected no publication bias, indicating that the pooled result is reliable.

## Conclusions

The evidence from the present meta-analysis supports the notion that both the rs12203592 and rs872071 IRF4 gene polymorphisms are associated with an individual’s susceptibility to skin cancer and haematological malignancies. The effect of the rs12203592 polymorphism on cancer is particularly prominent among Caucasians; however, no significant association with cancer risk was demonstrated in the NHL subgroup. Based on the limitations of the present study listed above, further functional studies between these polymorphisms and cancer risk are warranted.

## Abbreviations

IRF4: Interferon regulatory factor 4; HL: Hodgkin lymphoma; NHL: Non-Hodgkin lymphoma; MM: Multiple myeloma; CLL: Chronic lymphocytic leukaemia; BCC: Basal cell carcinoma; SCC: Squamous cell carcinoma; PB: Population based; HB: Hospital based; OR: Odd ratio; CI: Confidence interval.

## Competing interests

The authors declare that they are no competing interests.

## Authors’ contributions

SW, QY participated in collection of data and manuscript preparation. SW, QY and PC performed the statistical analysis. AG, PZ, PC participated in study design and critically revised the manuscript. AG and PZ participated in study design and manuscript preparation. All authors read and approved the final manuscript.

## Pre-publication history

The pre-publication history for this paper can be accessed here:

http://www.biomedcentral.com/1471-2407/14/410/prepub
